# Penetration and Adaptation of the Highly Viscous Zinc-Reinforced Glass Ionomer Cement on Contaminated Fissures: An In Vitro Study with SEM Analysis

**DOI:** 10.3390/ijerph19106291

**Published:** 2022-05-22

**Authors:** Galiah Husam AlJefri, Sunil Babu Kotha, Muhannad Hani Murad, Reham Mohammed Aljudaibi, Fatmah Nasser Almotawah, Sreekanth Kumar Mallineni

**Affiliations:** 1 Private Practitioner, Pediatric Dentistry, Riyadh 11614, Saudi Arabia; galiah.aljefri@gmail.com; 2 Pediatric Dentistry Division, Preventive Dentistry Department, College of Dentistry, Riyadh Elm University (REU), Riyadh 13244, Saudi Arabia; rmaljudaibi@moh.gov.sa (R.M.A.); fatmah.almotawah@riyadh.edu.sa (F.N.A.); 3 Department of Pediatric and Preventive Dentistry, Sharad Pawar Dental College and Hospital, Datta Meghe Institute of Medical Sciences, Sawangi (Meghe), Wardha 442004, Maharashtra, India; 4 Prince Salman bin Muhammad Hospital, Ministry of Health, Ad Dilam Governorate, Riyadh 16223, Saudi Arabia; dr.muhannadhm@hotmail.com; 5 Dental Public Health, College of Dentistry, Riyadh Elm University (REU), Riyadh 13244, Saudi Arabia; 6 Pediatric Dentistry Specialist, Al Muzahmiyah General Hospital, Ministry of Health, Al Muzahmiyah, Riyadh 11972, Saudi Arabia; 7 Department of Preventive Science, College of Dentistry, Majmaah University, Almajmaah 11952, Saudi Arabia; 8 Center for Transdisciplinary Research (CFTR), Saveetha Dental College, Saveetha Institute of Medical and Technical Sciences, Saveetha University, Chennai 600077, India

**Keywords:** glass ionomer cement, fissures, zinc, children, fluoride

## Abstract

Objective: We evaluate the penetration and adaptation of highly viscous zinc-reinforced glass ionomer cement (ZRGIC), using a scanning electron microscope (SEM), when applied under various contaminated conditions on grooves and fissures of primary second molars. Materials and Methods: A total of 40 extracted human primary second molars were randomly assigned into five groups (8 teeth each), with different surface conditions (conditioned with 40% polyacrylic acid, dry condition, water contamination, saliva contamination, or saliva contamination and air-drying) on the occlusal surface before placement of zinc-reinforced highly viscous glass ionomer cement with the finger-press technique. After sectioning the teeth, they were subjected to SEM analysis, where four in each group underwent aging by thermocycling and the other four were without aging. ANOVA tests, post hoc analysis, and unpaired t-tests were used for statistical analyses. Results: There was a significant statistical difference in the sealant penetration in the non-aging group, but in the aging group, there was no significant statistical difference in the sealant penetration. On other hand, a significant statistical difference was found in the adaptation between all the groups (*p* < 0.05). Highly viscous zinc-reinforced glass ionomer fissure sealants have better fissure penetration and more intimate adaptation under fissures conditioned with 40% polyacrylic acid and dry surface fissures with no contamination. However, the best penetration and retention after aging were under contaminated fissures with a shiny layer of saliva. Conclusions: Based on this study, we conclude that ZRGIC, a highly viscous fluoride-releasing cement, effectively seals fissures by interfering with food lodgment and protecting teeth from caries. We also conclude from this research that although the contaminated surfaces are not fully effective in penetrating and adapting the GIC to the tooth surface, they are still adequate for the brief period that will delay the carious process. It is advisable to restore the fissures with the minimal technique of sensitive fluoride-releasing GIC, particularly in young, uncooperative children, rather than leaving a caries-prone environment.

## 1. Introduction

Dental caries is defined as an infectious microbiologic disease, caused by an ecological shift in the composition and activity of the bacterial biofilm when exposed over time to fermentable carbohydrates, leading to a break in the balance between demineralization and remineralization. Carious lesions are preventable by averting onset and are manageable by implementing interventions [1]. Worldwide, it is considered the most common oral health disease in young children [2]. The cause of it is multifactorial: various factors can affect its occurrence such as malnutrition, genetic predisposition, poor health performance, specific eating habits, the presence of organisms affecting tooth decay such as streptococci, fluoride deficiency, vitamin D deficiency, low saliva flow rates, developmental defects of tooth enamel, maternal caries, high maternal levels of cariogenic bacteria, poor maternal oral hygiene, excessive sugar consumption, and prolonged bottle feeding; in addition, age, gender, and place of residence of children impact tooth decay. The importance of the primary teeth should not be overlooked, because, as has been said, healthy teeth in childhood have an important role in the emergence of healthy permanent teeth, healthy nutrition, and one’s aesthetic appearance [3,4]. In addition, we must consider that these teeth are particularly critical because even following repair, the affected tooth structure exhibits increased vulnerability to damage [5]. It has been noticed that children with dental caries are exposed to fear and anxiety, which can result in both severity and incomplete treatment of the condition [2,6].

Currently, the practice in modern dental clinics in many countries, especially for treating uncooperative patients, the elderly, and special needs patients, involves atraumatic restorative treatment (ART). This approach has developed and engendered considerable interest worldwide, not only in developing countries where resources are not readily available and affordable, but also increasingly in more industrialized countries [7,8]. Sealing of caries-prone pits and fissures with a sealant as a prevention method is based on one of the two ART approaches [7,8,9]. The placement of pit and fissure sealants is considered an effective modality; they act as physical barriers that isolate the covered areas of teeth from the oral environment, thereby preventing dental plaque accumulation and caries onset on occlusal surfaces of posterior teeth, and arrest caries progression [10,11,12]. According to the ART method, fissures that are at high risk of developing a carious lesion and those that have already developed an enamel carious lesion are indications for placing a sealant [7,8]. Molars may have more risk for caries, due to the complex shape of their occlusal fissure morphology, which is considered an ideal site for the retention of bacteria and food remnants and is inaccessible to mechanical cleaning/debridement [12]. Effectively penetrating and sealing these surfaces with a dental material can prevent lesions and is part of a comprehensive caries management approach [1].

Various materials and techniques used as pit and fissure sealants are available in the market [13]. There are four types of sealant materials under a classification proposed by Anusavice and colleagues: resin-based sealants, glass ionomer (GI) cements, GI sealants, polyacid-modified resin sealants, and resin-modified GI sealants [1,14,15,16]. Since the introduction of glass ionomer materials, they have been successfully employed for a number of applications [17]. Interest in their use as fissure sealants has been stimulated. It has been shown that glass ionomer fissure sealants efficaciously prevent occlusal caries, and the effectiveness of GIC, when placed as a pit and fissure sealant using the finger-press method, is an important aspect of caries management [10]. Some inherent physical and chemical properties make GIC an excellent dental restorative material in selected clinical situations. These properties include the prolonged release of fluoride and production of antibacterial action, chemical bonding to enamel and dentine, biocompatibility with pulpal tissue, and a coefficient of thermal expansion that is slightly lower than that of tooth structure [10,12,18,19]. Despite their outstanding properties, GICs have some disadvantages, including inadequate retention, lack of toughness, early water sensitivity, and low abrasion resistance. The main drawback of GIC when used is its relatively poor strength [19,20]. To address this concern, several “high-strength” GICs have been developed [21], such as zinc-reinforced glass ionomer (ZRGI) restorative material (ChemFil Rock, Dentsply Caulk), which was introduced to enhance mechanical properties, such as flexural strength, hardness, wear-resistance, and fracture toughness, instead of traditional resin or metal additives [20,22]. Another property of zinc is that it accumulates in the surface structures of teeth. Concentrations of zinc range from 430 to 2100 ppm in the surface enamel of teeth from different areas. In enamel, the major deposition of zinc takes place before tooth eruption. However, post-eruptive deposition of zinc appears to be irregular. Zinc is readily acquired by synthetic hydroxyapatite, competing with calcium for positions on the surface of the apatite crystal. Zinc pretreatment of hydroxyapatite produces resistance to acid dissolution [23]. There are many discrepancies among manufacturers’ instructions concerning the handling and use of GIC. Some suggest that dentine surfaces should be conditioned with polyacrylic acid prior to cement placement, while others, such as the World Health Organization (WHO), recommend the dilution of the acid with water in order to remove the dentin smear layer [24,25].

It is generally accepted that the effectiveness of sealants depends on long-term retention [11]. The long-term results of sealant retention are still controversial. It has been reported that approximately 50% of the applied sealant volume is lost after 1 month, followed by 75% at the end of 2 years [19]. Variations in sealant retention among different sealant systems might be related to many factors. These factors may include some technical errors such as salivary contamination, material characteristics and fissure morphology, material penetration into fissures, and material adaptability to the fissure walls [18,19]. Other possible reasons for this early loss include the presence of organic debris, wear or fracture of sealant materials or unetched areas after routine cleaning, the physical and chemical properties of the enamel, effects of thermal changes, and the clinical technique. An optimal sealant adaptation is necessary to prevent marginal microleakage. Penetration of the sealant into the complete depths of pits and fissures, its lateral wall adaptation, and subsequent retention are the key factors in the longevity of these restorations [12]. Therefore, the marginal sealing ability of sealing materials is extremely important for successful treatment [19].

In children who are uncooperative in the dental clinic, it is difficult to ensure an isolated environment during their treatment, but we cannot leave the caries-prone tooth surfaces exposed to sugars and thus worsen the condition [15,16]. Zinc is one of the contributing factors in post-eruptive mineralization, so we considered zinc-containing GIC. There is little evidence in the literature regarding use of highly viscous zinc-reinforced glass ionomer cement as a fissure sealant in contaminated fissures. Thus, we aimed to evaluate the penetration and adaptation of highly viscous zinc-reinforced glass ionomer cement (ZRGIC) using a scanning electron microscope (SEM) when applied under various contaminated conditions on grooves and fissures of primary second molars. The null hypothesis of this study was that there is no statistical difference in penetration and adaptation of highly viscous zinc-reinforced glass ionomer cement in different dried and moist contaminated surfaces.

## 2. Methods

### 2.1. Sample Size Calculation

**Figure 1:**
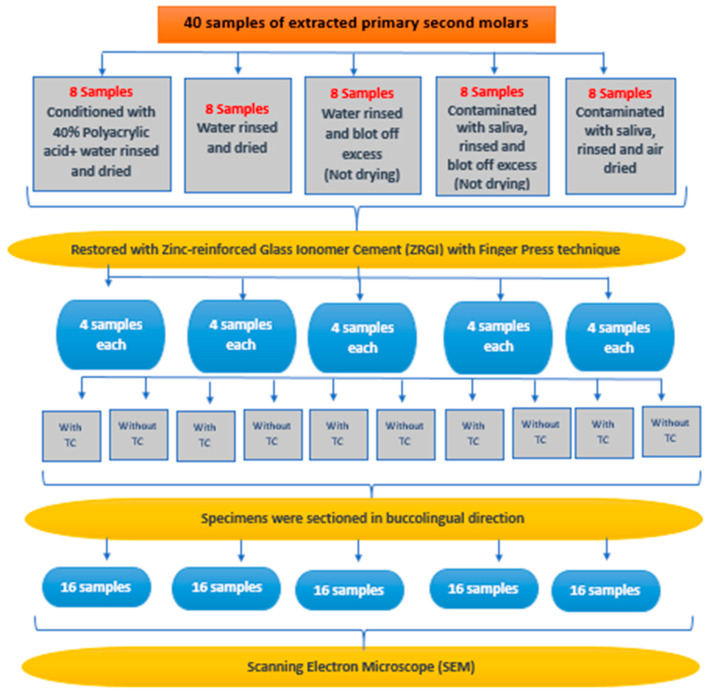
Sample distribution TC: thermocycling).

The sample size was estimated using GPower 3.0.10 software [26]. The effect size of 0.8 was calculated from the data of a similar study [18]. The alpha error was fixed at 5%, and the beta error was set at 20%. Therefore, the power of the study was 80%. The minimum sample size estimated per group was 11 samples. Therefore, the total sample size was 55 for five groups. In our study, each tooth was sectioned, and we obtained 2 samples from each tooth; thus, from the 40 teeth used in the study, 80 samples were prepared, distributed with 16 per group (Figure 1).

- *t*-tests—means: difference between two independent means (two groups).
- Analysis—a priori: compute required sample size.
- Input—tail(s) = 1; effect size d = 1.1030078; α err prob = 0.05; power (1-β err prob) = 0.8; allocation ratio N2/N1 = 1.
- Output—no centrality parameter δ = 2.586783; critical *t* = 1.724718; df = 20; sample size per group = 11; actual power = 0.803133.

### 2.2. Sample Collection

A total of 40 extracted human primary second molars were used in this study. Teeth were cleaned with water/pumice slurry using a dental prophylactic cup, and then they were stored in distilled water. The teeth were then randomly assigned into five groups (eight teeth each). The combination of five surface conditions defined the treatment groups.

### 2.3. Preparation of the Occlusal Surfaces

**Figure 2:**
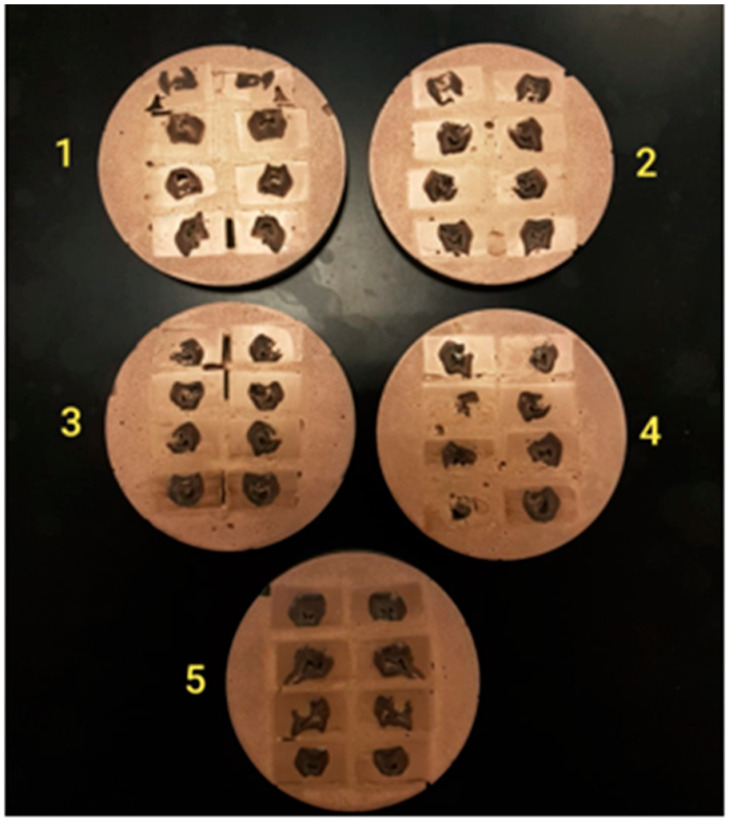
Samples sectioned buccolingual and gold-sputtered.

Each group was divided according to the following different surface conditions before the placement of zinc-reinforced highly viscous glass ionomer cement (Figure 2):

Group 1: Occlusal surfaces were conditioned with 40% polyacrylic acid, rinsed with water for 10 s, and then dried with no contamination.

Group 2: Occlusal surfaces were rinsed with water and dried with no contamination.

Group 3: A drop of water was syringed onto the enamel’s occlusal surface and left undisturbed for 10 s. The excess water was then blotted with a small sponge, leaving a moist, shiny enamel surface.

Group 4: A drop of fresh human saliva was syringed onto the enamel’s occlusal surface and left undisturbed for 10 s. The excess saliva was then blotted with a small sponge, leaving a moist, shiny enamel surface.

Group 5: A drop of fresh human saliva was syringed onto the enamel’s occlusal surface for 10 s, then the surface was air-dried for 5 s [18].

### 2.4. Sample Distribution

#### Sealant Application

The zinc-reinforced glass ionomer (ZRGI) sealant material (ChemFil Rock, Dentsply Caulk) was manipulated according to the manufacturer’s instructions. It was triturated for 10 s, and then applied with pressure using a gloved finger to the occlusal surface of the tooth, overfilling it slightly. After that, the restoration was condensed with the finger-press technique, and the occlusion was checked [10]. Excess material was removed with a carver or a flat plastic instrument, and the bite was readjusted if necessary, making sure that the occlusal fissures were sealed. Vaseline was applied over the restoration to protect the glass ionomer during the initial setting reaction. After sealing the fissures, all 8 teeth from each group (Group 1–5) were subdivided into non-aging and aging groups (Figure 1). Non-aging group samples were prepared for SEM analysis. The aging group was subjected to thermocycling for 10,000 cycles at 5 and 55 °C with a dwell time of 60 s in each bath and a transfer time of 3 s [27,28,29]. Prior to this, it was embedded in epoxy resin to stabilize the specimens during the procedure, and then subjected to SEM analysis.

**Figure 3:**
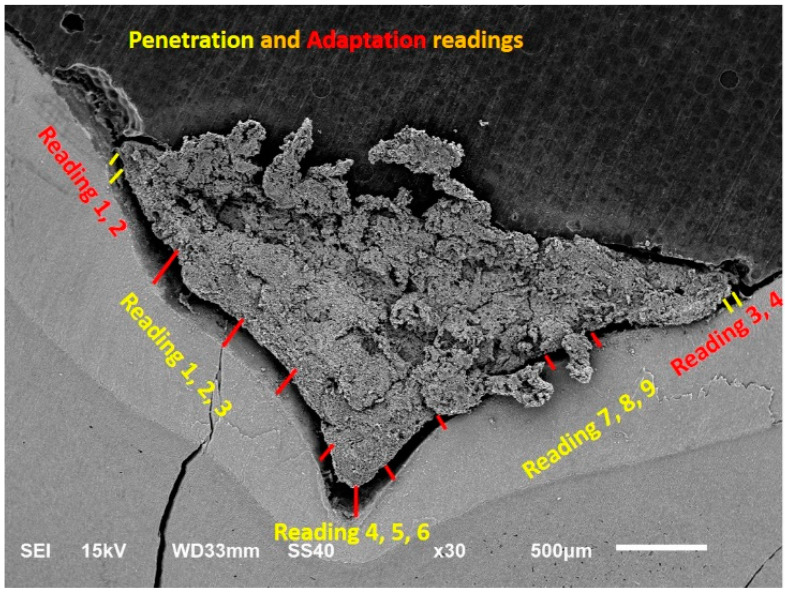
Nine different areas of measurement for penetration, and four different areas of measurements for adaptation (kV = kilovolts, mm = millimeters, µm = micro meters).

The root portions of all teeth in the non-aging and aging groups (40 teeth) were cut off, and then the crown portions were mounted on acrylic blocks covering the whole crown. Then, they were sectioned buccolingually with a water-cooled diamond saw (Precision Saw, Isomet 2000/BUEHLER, Lake Bluff, IL, USA) achieving 16 samples in each group (Group 1–5) (Figure 3). All specimens were allowed to dry for 24 h, after which they were mounted on aluminum stubs using double-sided adhesive tape; they were mounted in a way that the area to be studied faced upward. The mounting surfaces were then sputtered with a thin layer (25 nm thickness) of pure gold using an ion sputtering unit. Later, the aluminum stubs were placed in the vacuum chamber of the SEM. The accelerating voltage, angle of tilt, and the aperture were adjusted to optimize the quality of the micrograph and to suit the specimens. The surfaces were then scanned and observed on the screen under different magnifications (×13 to ×1500).

## 3. Data Analysis

The data analysis was performed using SPSS software to analyze any statistical difference in the penetration and adaptability of the zinc-containing, highly viscous GIC. Tukey’s test was used to test if there was any significant difference in the sealant penetration and adaptation values among the different groups. ANOVA was used to compare the penetration and adaptation of all the groups at the same time to determine whether a relationship existed among them. Post hoc analysis was used to analyze the variations in the depth and penetration of GIC within the groups and subgroups. An unpaired *t*-test was used to compare the averages/means of two unrelated groups to determine if there is a significant difference between them.

## 4. Results

The study sample comprised 40 extracted human primary second molars that were randomly divided into 8 teeth for each group (Groups 1–5) and then subdivided into 4 teeth per non-aging and aging group. Teeth in the non-aging group were immediately placed in the vacuum chamber of the SEM. Teeth in the aging group were subjected to thermocycling for 10,000 cycles in 5 and 55 °C, and then placed in the vacuum chamber of the SEM. The penetration and adaptation results of the highly viscous zinc-reinforced glass ionomer cement under different contamination conditions were tested and measurements were taken in µm. Nine measurements in different areas for penetration and four measurements in different areas for adaptation and the averages were taken, as shown in the data and tables that follow.

The penetration depth results of zinc-reinforced glass ionomer cement (ZRGIC) (ChemFil Rock, Dentsply Caulk) tested under different contamination conditions in the non-aging group are shown in Table 1. The mean and standard deviation values of the penetration depth of the groups were as follows: Group 1, conditioned with 40% polyacrylic acid, with no contamination, 1382 ± 923 µm; Group 2, occlusal surfaces rinsed with no contamination, 1527 ± 438 µm; Group 3, moist shiny occlusal surface contaminated with water, 654 ± 591 µm; Group 4, moist shiny enamel surface, contaminated with saliva, 455 ± 84 µm; Group 5, occlusal surface contaminated with saliva and dried, 458 ± 215 µm.

The adaptation results of zinc-reinforced glass ionomer cement (ZRGIC) (ChemFil Rock, Dentsply Caulk) tested under different contamination conditions in the non-aging group are shown in Table 2. The mean and standard deviation values of the penetration depth of the groups were as follows: Group 1, conditioned with 40% polyacrylic acid, with no contamination, 117 ± 50 µm; Group 2, occlusal surfaces rinsed with no contamination, 120 ± 58 µm; Group 3, moist shiny occlusal surface contaminated with water, 75 ± 39 µm; Group 4, moist shiny enamel surface, contaminated with saliva, 97 ± 53 µm; Group 5, occlusal surface contaminated with saliva and dried, 43 ± 28 µm.

The penetration depth results of zinc-reinforced glass ionomer cement (ZRGIC) (ChemFil Rock, Dentsply Caulk) tested under different contamination conditions in the aging group are shown in Table 3. The mean and standard deviation values of the penetration depth of the groups were as follows: Group 1, conditioned with 40% polyacrylic acid, with no contamination, 647 ± 322 µm; Group 2, occlusal surfaces rinsed with no contamination, 758 ± 234 µm; Group 3, moist shiny occlusal surface contaminated with water, 794 ± 243 µm; Group 4, moist shiny enamel surface, contaminated with saliva, 899 ± 471 µm; Group 5, occlusal surface contaminated with saliva and dried, 714 ± 279 µm.

The adaptation results of zinc-reinforced glass ionomer cement (ZRGIC) (ChemFil Rock, Dentsply Caulk) tested under different contamination conditions in the aging group are shown in Table 4. The mean and standard deviation values of the penetration depth of the groups were as follows: Group 1, conditioned with 40% polyacrylic acid, with no contamination, 2146 ± 962 µm; Group 2, occlusal surfaces were rinsed with no contamination, 2407 ± 590 µm; Group 3, moist shiny occlusal surface contaminated with water, 1517 ± 647 µm; Group 4, moist shiny enamel surface, contaminated with saliva, 1454 ± 427 µm; Group 5, occlusal surface contaminated with saliva and dried, 1221 ± 391 µm.

The penetration depth results of zinc-reinforced glass ionomer (ZRGI) (ChemFil Rock, Dentsply Caulk) fissure sealant tested for Group 1, where the occlusal surfaces were conditioned with 40% polyacrylic acid, then rinsed with water for 10 s then dried with no contamination, are shown in Table 5. The mean and standard deviation values of the penetration depth of the non-aging group were 1381.50 ± 923.139 µm, and those for the aging group were 647.13 ± 322.219 µm. There was no significant difference between the means of the two groups.

The penetration depth results of zinc-reinforced glass ionomer (ZRGI) (ChemFil Rock, Dentsply Caulk) fissure sealant tested for Group 2, where the occlusal surfaces were rinsed with water and dried with no contamination, are shown in Table 6. The mean and standard deviation values of the penetration depth of the non-aging group were 1527.00 ± 437.986 µm, and those for the aging group were 757.75 ± 234.191 µm. There was a significant difference between the means of the two groups, non-aging and aging.

The penetration depth results of zinc-reinforced glass ionomer (ZRGI) (ChemFil Rock, Dentsply Caulk) fissure sealant tested for Group 3, where a drop of water was syringed onto the occlusal surface of the enamel and left undisturbed for 10 s, and the excess water was then blotted with a small sponge, leaving a moist, shiny enamel surface, are shown in Table 7. The mean and standard deviation values of the penetration depth of the non-aging group were 645.50 ± 591.025 µm, and those for the aging group were 749.13 ± 242.788 µm. There was no significant difference between the means of the two groups, non-aging and aging.

The penetration depth results of zinc-reinforced glass ionomer (ZRGI) (ChemFil Rock, Dentsply Caulk) fissure sealant tested for Group 4, where a drop of fresh human saliva was syringed onto the occlusal surface of the enamel and left undisturbed for 10 s, and the excess saliva was then blotted with a small sponge, leaving a moist, shiny enamel surface, are shown in Table 8. The mean and standard deviation values of the penetration depth of the non-aging group were 454.50 ± 83.526 µm, and those for the aging group were 898.75 ± 470.987 µm. There was a significant difference between the means of the two groups, non-aging and aging.

The penetration depth results of zinc-reinforced glass ionomer (ZRGI) (ChemFil Rock, Dentsply Caulk) fissure sealant tested for Group 5, where a drop of fresh human saliva was syringed onto the occlusal surface of the enamel for 10 s, after which the surface was air-dried for 5 s, are shown in Table 9. The mean and standard deviation values of the penetration depth of the non-aging group were 458.13 ± 215.478 µm, and those for the aging group were 714.38 ± 279.171 µm. There was a significant difference between the means of the two groups, non-aging and aging.

An ANOVA test was performed to examine the penetration depth of zinc-reinforced glass ionomer cement (ZRGIC) (ChemFil Rock, Dentsply Caulk) under different contamination conditions in the non-aging group. Table 10 provides the mean and standard deviation values of the penetration depth of Group 1 (1381.50 ± 923.139 µm), Group 2 (1527.00 ± 438.986 µm), Group 3 (654.50 ± 591.025 µm), Group 4 (454.50 ± 83.526 µm), and Group 5 (458.13 ± 215.478 µm). There was a significant difference between the means of all the groups in the non-aging group.

The pairwise comparisons of the groups show that there was statistical variation in penetration in the non-aging group immediately after restoration (Table 11).

An ANOVA test was performed to examine the adaptation results of zinc-reinforced glass ionomer cement (ZRGIC) (ChemFil Rock, Dentsply Caulk) tested under different contamination conditions in the non-aging group. Table 12 contains the mean and standard deviation values of the penetration depth of Group 1 (117.00 ± 49.558 µm), Group 2 (119.63 ± 58.243 µm), Group 3 (74.75 ± 38.751 µm), Group 4 (97.25 ± 53.452 µm), and Group 5 (43.25 ± 28.454 µm). There was a significant difference between the means of all the groups in the non-aging group.

The pairwise comparisons of the groups show that there was statistical variation in adaptation in the non-aging group immediately after restoration (Table 13).

An ANOVA test was performed to examine the penetration depth results of zinc-reinforced glass ionomer cement (ZRGIC) (ChemFil Rock, Dentsply Caulk) tested under different contamination conditions in the aging group. Table 14 lists the mean and standard deviation values of the penetration depth of Group 1 (647.13 ± 322.219 µm), Group 2 (757.75 ± 234.191 µm), Group 3 (794.13 ± 242.788 µm), Group 4 (898.75 ± 470.987 µm), and Group 5 (714.38 ± 279.171 µm). There was no significant difference between the means of all the groups in the aging group.

The pairwise comparisons of the groups show that there was statistical variation in penetration in the aging group after thermocycling (Table 15).

An ANOVA test was performed to examine the adaptation results of zinc-reinforced glass ionomer cement (ZRGIC) (ChemFil Rock, Dentsply Caulk) tested under different contamination conditions in the aging group. Table 16 shows the mean and standard deviation values of the penetration depth of Group 1 (2146.38 ± 961.966 µm), Group 2 (2406.50 ± 589.933 µm), Group 3 (1517.25 ± 646.951 µm), Group 4 (1454.25 ± 427.085 µm), and Group 5 (1220.63 ± 391.174 µm). There was a significant difference between the means of all the groups in the aging group.

The pairwise comparisons of the groups show that there was statistical variation in adaptation in the aging group after thermocycling (Table 17).

There is a significant statistical difference in the sealant penetration in the non-aging group. However, in the aging group, there was no significant statistical difference in the sealant penetration. On other hand, a significant statistical difference was found in the adaptation between all the groups (*p* < 0.05). Therefore, the null hypothesis was rejected. Zinc-reinforced glass ionomer cement did not penetrate well into the fissures under the different conditions of contaminated fissures, but it adapted well.

## 5. Discussion

The preventive advantage of the pit and fissure sealants is only guaranteed when the sealant has been completely preserved with adequate adaptation to the enamel [30,31]. There are not enough studies in which highly viscous zinc-reinforced glass ionomer fissure sealants have been investigated. In the present study, penetration and adaptation of highly viscous zinc-reinforced glass ionomer fissure sealants were evaluated under different contamination conditions using SEM analysis. The use of SEM, owing to its magnification and depth of focus, provides a means of direct visual observation of penetration and adaptation of sealant materials to enamel walls. In the present study, numerical measurements were used rather than rating score systems in the assessment of sealant penetration and adaptation. The computer software that calculates the measurements gives better results than manual calculation using a scoring system.

In this study, thermal cycling was also performed in order to simulate temperature variations that occur daily in the oral cavity. There was a significant difference in adaptation between the means of all the non-aging and aging groups. The sealant penetration of highly viscous zinc-reinforced glass ionomer fissure sealants showed better results under conditioned fissures with 40% polyacrylic acid and dry surface with no contamination but did not last for a long period. In addition, the penetration depth results of zinc-reinforced glass ionomer cement (ZRGIC) (ChemFil Rock, Dentsply Caulk) tested under different contamination conditions showed no significant difference between the means of all the groups in the aging group. All these findings correlate with Titley et al. [32], who reported that the effect of thermal cycling did not alter the bond strength of the materials to the enamel. Koyuturk et al. [33] applied a low number of thermal cycles (10,000 times) to specimens, and they had no influence on microleakage. In this way, the effect of thermocycling on microleakage was barred and precluded. The high mean values of adaptation that were found in Group 1, where occlusal surfaces were conditioned with 40% polyacrylic acid, and Group 2, where occlusal surfaces were rinsed with water and dried with no contamination, are because the absence of water in the enamel can be a favorable factor for the durability of bond strength after thermal cycling since its presence in the substrate can facilitate water absorption by the adhesive, allowing hydrolysis at the adhesive interface after thermal cycles, damaging the bond strength [34].

The findings of this study indicate that there was a negative effect of the dried saliva contamination on the adaptation and penetration of highly viscous zinc-reinforced glass ionomer fissure sealants in the short period, but after aging, the penetration was the best under a contaminated surface with a shiny layer of saliva. The results of Al-Jobair et al. [18] indicated that there was no negative effect of the dried saliva contamination on the penetration and adaptation of fissure sealant. In addition, a study by Thomson et al. [35] indicated that successful sealing may in fact be possible following salivary contamination, provided the enamel is washed thoroughly within a short time of contamination. Moreover, the main finding of a study by Shimazu et al. [36] was that artificial saliva contamination did not affect the adhesion of GIC and RMGIC. The results of the study suggest that GIC and RMGIC are suitable for restorative treatment when isolation using a rubber dam is not feasible [36]. However, these results conflict with the conclusions of Meurman, who reported that salivary contamination of the enamel surface must have a detrimental effect on sealant retention in vivo [35]. Chen demonstrated that saliva contamination lowered the bond strength between GIC and enamel surface [37].

Polyacrylic acid is usually used to enhance the adhesion of cement to the enamel surface by intercrystalline bonding in addition to calcium complexation and hydrogen bonding; however, the efficiency of this material is inconsistent, as no crystalline formation on the enamel surface was found under low-vacuum SEM. On the contrary, a pitted enamel surface was produced, which may result in weak bonding due to poor penetration of highly viscous GIC into pits. In addition, the acid retained on the surface without rinse-off may form a gel that hinders the GIC from bonding to the enamel. Our results show the suitable performance of highly viscous zinc-reinforced glass ionomer fissure sealants regarding adaptation to the fissure walls under conditioned fissures with 40% polyacrylic acid and a dry surface with no contamination even after aging, approximately resembling a 1-year period. Frencken et al. [9] suggested washing the polyacrylic acid-conditioned tooth surface with a water-moistened cotton pellet several times and then drying with dry pellets before filling GIC into the cavity. This washing procedure may create a relatively clean enamel surface, which would result in more favorable contact between GIC and the enamel surface [37].

Nevertheless, the results of Al-Jobair’s study propose that resin-based fissure sealant can be used in a moisture-controlled environment [18]. Highly viscous zinc-reinforced glass ionomer fissure sealants may provide effective sealants in the treatment of young or uncooperative children and children with special needs who are unable to follow meticulous isolation methods. Moreover, it can be used in the treatment of partially erupted teeth that are difficult to isolate and in situations where a “transitional” sealant may be considered before the placement of a “permanent” resin sealant [18]. Muntean et al. [38] concluded that resin-modified glass ionomer sealant could be used as a transitional sealant in specific conditions, especially in uncooperative patients with high caries risk, even if the mechanical properties of this material did not reach the accomplishment of resin-based sealant.

Limitations were the preservation of test specimens in distilled water and the employment of thermocycling with artificial saliva to simulate the oral environment. Justus et al. [39] mentioned that when tooth specimens are stored in distilled water, the organic content of the enamel surface may be partially lost. Therefore, our results regarding enamel deproteinization might be slightly inflated compared to those previously reported. On the other hand, Harleen et al. [40] reported that in vitro tests do not completely predict how dental materials will behave in the oral cavity. In spite of the limitations, using thermocycling in our study was convenient, and according to the International Organization for Standardization, thermocycling is the best process for mimicking thermal changes in the oral environment during in vitro studies [41]. In all, the study provides important information to encourage additional clinical research on the use of highly viscous zinc-reinforced glass ionomer fissure sealants in children. The results of the study must be observed along with some limitations, as it is an in vitro study. In vitro studies are useful to explain some conditions of materials separate from when they are exposed in the oral cavity [42,43]. Therefore, the results cannot be extrapolated to clinical practice, since multiple factors are acting simultaneously on the dental materials. Another limitation present in the current study is the preservation of test specimens in distilled water. Justus et al. [39] mentioned that when tooth specimens are stored in distilled water, the organic content of the enamel surface may be partially lost. Therefore, our results regarding enamel deproteinization might be slightly inflated. Furthermore, Harleen et al. [40] reported that in vitro tests do not completely predict how dental materials will behave in the oral cavity [41]. An Indian study [44] concluded that classical sealant was the best compared to flowable nanocomposite for both penetration and microleakage properties. The authors used 15 samples in their study. The present study was not compared because the authors compared adaptation and penetration of ZRGIC with 16 samples in a group. Prior studies [45,46,47] reported with mixed results using the penetration of sealants into fissures and the present study used ZRGIC, hence the present study findings were not comparable with those studies. Another limitation is that the authors did not consider the type of fissure morphology that makes a difference in penetration and adaptation. Lastly, the authors did not take into account hypoplastic conditions (e.g., amelogenesis imperfecta) to assess the penetration and adaptation. The present study was planned to restore the contaminated tooth surfaces and analyze the restorative material’s adaptation and penetration ability. Based on the results described above, the authors conclude that ZRGIC, a highly viscous fluoride-releasing cement, effectively seals fissures by interfering with food lodgment and protecting teeth from caries. The authors also agree in this research that although the contaminated surfaces are not fully effective in penetrating and adapting the GIC to the tooth surface, they are adequate for the brief period that will delay the carious process. It is advisable to restore the fissures with a minimal technique using sensitive fluoride-releasing GIC, particularly in young, uncooperative children, rather than leaving a caries-prone environment. There is a need for clinical trial studies to investigate the long-term retention of highly viscous zinc-reinforced glass ionomer fissure sealants. Future studies in vivo can include follow-up intervals of 6 months, 1 year, and 2 years. Studies might consider investigating the effect of the fissure morphology together with the adaptation and penetration.

## 6. Conclusions

Based on the results of this study, and within its limitations, the following conclusions can be made:

1. Highly viscous zinc-reinforced glass ionomer fissure sealants have better fissure penetration and more intimate adaptation under fissures conditioned with 40% polyacrylic acid and dry surface fissures with no contamination.
2. Highly viscous zinc-reinforced glass ionomer fissure sealants have the best penetration and retention after aging under contaminated fissures with a shiny layer of saliva.
3. Sealant penetration and adaptation are influenced by the type of fissure surface contamination.

## Figures and Tables

**Table 1:** Penetration depth of ZRGIC in the non-aging group of different treatment groups.

		Penetration Depth: Non-Aging Group (µm)	
		Mean	Standard Deviation
Group	1	1382	923
	2	1527	438
	3	645	591
	4	455	84
	5	458	215

**Table 2:** Adaptation of ZRGIC in the non-aging group of different treatment groups.

		Adaptation in Non-Aging Group (µm)	
		Mean	Standard Deviation
Group	1	117	50
	2	120	58
	3	75	39
	4	97	53
	5	43	28

**Table 3:** Penetration depth of ZRGIC in the aging group of different treatment groups.

		Penetration Depth in Aging Group (µm)	
		Mean	Standard Deviation
Group	1	647	322
	2	758	234
	3	794	243
	4	899	471
	5	714	279

**Table 4:** Adaptation of ZRGIC in the aging group of different treatment groups.

		Adaptation in Aging Group (µm)	
		Mean	Standard Deviation
Group	1	2146	962
	2	2407	590
	3	1517	647
	4	1454	427
	5	1221	391

**Table 5:** The penetration depth of ZRGIC in Group 1 (non-aging and aging) (µm).

		Mean	*n*	Std. Deviation	*t* Value	*p* Value
Group = 1	Penetration Depth: Non-Aging Group	1381.50	8	923.139	2.087	0.075
	Penetration Depth Aging Group	647.13	8	322.219		

Unpaired *t*-test, statistical significance at *p* ≤ 0.05.

**Table 6:** Penetration depth of ZRGIC in Group 2 (non-aging and aging) (µm).

		Mean	*n*	Std. Deviation	*t* Value	*p* Value
Group = 2	Penetration Depth: Non-Aging Group	1527.00	8	437.986	5.981	0.001 *
	Penetration Depth Aging Group	757.75	8	234.191		

Unpaired *t*-test, * statistical significance at *p* ≤ 0.05.

**Table 7:** The penetration depth of ZRGIC in Group 3 (non-aging and aging) (µm).

		Mean	*n*	Std. Deviation	*t* Value	*p* Value
Group = 3	Penetration Depth: Non-Aging Group	645.50	8	591.025	−0.684	0.516
	Penetration Depth Aging Group	794.13	8	242.788		

Unpaired *t*-test, statistical significance at *p* ≤ 0.05.

**Table 8:** The penetration depth of ZRGIC in Group 4 (non-aging and aging) (µm).

		Mean	*n*	Std. Deviation	*t* Value	*p* Value
Group = 4	Penetration Depth: Non-Aging Group	454.50	8	83.526	−2.491	0.042 *
	Penetration Depth Aging Group	898.75	8	470.987		

Unpaired *t*-test, * statistical significance at *p* ≤ 0.05.

**Table 9:** Penetration depth of ZRGIC in Group 5 (non-aging and aging) (µm).

		Mean	*n*	Std. Deviation	*t* Value	*p* Value
Group = 5	Penetration Depth: Non-Aging Group	458.13	8	215.478	−2.459	0.044 *
	Penetration Depth Aging Group	714.38	8	279.171		

Unpaired *t*-test, * statistical significance at *p* ≤ 0.05.

**Table 10:** Penetration depth of ZRGIC in the non-aging group for all the groups (µm).

		Mean	Std. Deviation	F Value	*p* Value
Penetration Depth: Non-Aging Group	Group 1	1381.50	923.139	7.487	0.001 *
	Group 2	1527.00	437.986		
	Group 3	645.50	591.025		
	Group 4	454.50	83.526		
	Group 5	458.13	215.478		

ANOVA test, * statistical significance at *p* ≤ 0.05.

**Table 11:** Pairwise comparison of penetration depth of ZRGIC in the non-aging group within all groups (1–5) (µm).

	Group	Compared Group	Mean Difference	*p* Value
Penetration Depth Non-Aging Group	1	2	−145.500	0.982
		3	736.000	0.068
		4	927.000	0.012 *
		5	923.375	0.013 *
	2	3	881.500	0.019 *
		4	1072.500	0.003 *
		5	1068.875	0.003 *
	3	4	191.000	0.953
		5	187.375	0.956
	4	5	−3.625	1.000

Post hoc Tukey test; * statistical significance at *p* ≤ 0.05.

**Table 12:** Adaptation of ZRGIC in the non-aging group for all the groups (µm).

		Mean	Std. Deviation	F Value	*p* Value
Adaptation Non-Aging Group	Group 1	117.00	49.558	3.700	0.013 *
	Group 2	119.63	58.243		
	Group 3	74.75	38.751		
	Group 4	97.25	53.452		
	Group 5	43.25	28.454		

ANOVA test, * statistical significance at *p* ≤ 0.05.

**Table 13:** Pairwise comparison of adaptation of ZRGIC in the non-aging group within all groups (1–5) (µm).

	Group	Compared Group	Mean Difference	*p* Value
Adaptation Non-Aging Group	1	2	−2.625	1.000
		3	42.250	0.390
		4	19.750	0.916
		5	73.750	0.026 *
	2	3	44.875	0.330
		4	22.375	0.874
		5	76.375	0.020 *
	3	4	−22.500	0.872
		5	31.500	0.667
	4	5	54.000	0.169

Post hoc Tukey test, * statistical significance at *p* ≤ 0.05.

**Table 14:** Penetration depth of ZRGIC in the aging group for all the groups (µm).

		Mean	Std. Deviation	F Value	*p* Value
Penetration Depth: Aging group	Group 1	647.13	322.219	0.681	0.610
	Group 2	757.75	234.191		
	Group 3	794.13	242.788		
	Group 4	898.75	470.987		
	Group 5	714.38	279.171		

ANOVA test, statistical significance at *p* ≤ 0.05.

**Table 15:** Pairwise comparison of penetration depth of ZRGIC in the aging group within all groups (1–5) (µm).

	Group	Compared Group	Mean Difference	*p* Value
Penetration Depth Aging Group	1	2	−110.625	0.958
		3	−147.000	0.890
		4	−251.625	0.529
		5	−67.250	0.993
	2	3	−36.375	0.999
		4	−141.000	0.904
		5	43.375	0.999
	3	4	−104.625	0.965
		5	79.750	0.987
	4	5	184.375	0.781

Post hoc Tukey test.

**Table 16:** Adaptation of ZRGIC in the aging group for all the groups (µm).

		Mean	Std. Deviation	F Value	*p* Value
Adaptation: Aging Group	Group 1	2146.38	961.966	4.982	0.003 *
	Group 2	2406.50	589.933		
	Group 3	1517.25	646.951		
	Group 4	1454.25	427.085		
	Group 5	1220.63	391.174		

ANOVA test; * statistical significance at *p* ≤ 0.05.

**Table 17:** Pairwise comparison of adaptation of ZRGIC in the aging group within all groups (1–5) (µm).

	Group	Compared Group	Mean Difference	*p* Value
Penetration Depth Aging Group	1	2	−260.125	0.924
		3	629.125	0.298
		4	692.125	0.213
		5	925.750	0.046 *
	2	3	889.250	0.060 *
		4	952.250	0.038 *
		5	1185.875	0.006 *
	3	4	63.000	1.000
		5	296.625	0.883
	4	5	233.625	0.947

Post hoc Tukey test; * statistical significance at *p* ≤ 0.05.

## Data Availability

The data will be available upon request to correspondence authors.
